# Alcohol consumption, depression, overweight and cortisol levels as determining factors for *NR3C1* gene methylation

**DOI:** 10.1038/s41598-021-86189-z

**Published:** 2021-03-24

**Authors:** Júlia de Assis Pinheiro, Flávia Vitorino Freitas, Aline Ribeiro Borçoi, Suzanny Oliveira Mendes, Catarine Lima Conti, Juliana Krüger Arpini, Tamires dos Santos Vieira, Rafael Assis de Souza, Dirceu Pereira dos Santos, Wagner Miranda Barbosa, Anderson Barros Archanjo, Mayara Mota de Oliveira, Joaquim Gasparini dos Santos, Bruna Pereira Sorroche, José Claudio Casali-da-Rocha, Leonardo Oliveira Trivilin, Elizeu Batista Borloti, Iuri Drumond Louro, Lidia Maria Rebolho Batista Arantes, Adriana Madeira Alvares-da-Silva

**Affiliations:** 1 Biotechnology/Renorbio Graduate Program, Universidade Federal do Espirito Santo, Vitoria, ES Brazil; 2 Department of Pharmacy and Nutrition, Universidade Federal do Espirito Santo, Alegre, ES Brazil; 3 Graduate Program in Forest Sciences, Universidade Federal do Espirito Santo, Alegre, ES Brazil; 4 Department of Agronomia, Universidade Federal do Espirito Santo, Alegre, ES Brazil; 5 Fluminense Federal Institute, Campos dos Goytacazes, RJ Brazil; 6 Molecular Oncology Research Center, Hospital do Câncer de Barretos, Barretos, SP Brazil; 7 A. C. Camargo Cancer Center, São Paulo, SP Brazil; 8 Department of Medicina Veterinária, Universidade Federal do Espirito Santo, Alegre, ES Brazil; 9 Department of Social and Developmental Psychology, Universidade Federal do Espírito Santo, Vitoria, ES Brazil; 10 Department of Morphology, Health Science Center, Universidade Federal do Espirito Santo, Vitoria, ES Brazil; 11 Departamento de Biologia, Universidade Federal do Espirito Santo, Alto Universitário Sem Número, Alegre, ES 29500000 Brazil

**Keywords:** Epigenetics and behaviour, DNA methylation

## Abstract

The *NR3C1* glucocorticoid receptor (GR) gene is a component of the stress response system, which can be regulated by epigenetic mechanisms. *NR3C1* methylation has been associated with trauma and mental issues, including depression, post-traumatic stress, anxiety, and personality disorders. Previous studies have reported that stressful events are involved in *NR3C1* gene methylation, suggesting that its regulation under environmental effects is complex. The present study aimed to analyze associations involving stressors such as socioeconomic status, health conditions, and lifestyle in relation to *NR3C1* methylation in adults. This study included 386 individual users of the Brazilian Public Unified Health System (SUS), and evaluated socioeconomic and health conditions, body mass index, cortisol levels, and lifestyle. Data were correlated with *NR3C1* methylation, determined using DNA pyrosequencing. The results showed that alcohol consumption, overweight, and high cortisol levels were related to *NR3C1* demethylation, while depression was related to its methylation. Habits, lifestyle, and health status may influence *NR3C1* gene regulation via methylation, revealing the complexity of environmental impacts on *NR3C1* methylation.

## Introduction

DNA methylation is a widely known mechanism involved in regulating gene expression^1^. It has been recently described as the "*modus operandi*" of environmental adaptation, and in rapid responses to exposure events, which can be passed on to future generations^2^. Imprinting patterns are inherited and preserved during cell division; however, extrinsic or environmental factors contribute to epigenetic changes during an individual's life^3–5^. Therefore, stressful events can result in the addition or withdrawal of epigenetic marks at specific DNA locations, resulting in altered gene expression^6–8^.

Stress events in humans or animal models have been related to epigenetic changes in specific regulatory regions of the glucocorticoid receptor (GR) gene encoding, which has the function of regulating hypothalamic stress on the neuroendocrine hypothalamic–pituitary–adrenal (HPA) axis, via cortisol production^9,10^. Increased cortisol levels have been previously related to stress and methylation^11–13^. Furthermore, other stressful events or conditions, including trauma^14^, early life stress^13,15–17^, depression^10,18,19^, nutritional alterations^20–22^, psychosocial stress^23^, and consumption of substances such as alcohol and tobacco^24,25^, can alter GR gene encoding methylation levels.

Animal studies have evaluated methylation events directly in the hypothalamus^15,26^, while human studies have evaluated blood methylation events by their homology observed in different tissues with equivalent expression^16^.

The GR belongs to the ligand-dependent nuclear receptor transcription factor superfamily and, in humans, it is encoded by the *NR3C1* gene, located on chromosome 5q31–q32, with approximately 140,000 base pairs^27–29^. This gene is composed of 17 exons, eight coding exons (numbered 2–9), and nine non-coding exons, which are located in the gene promoter^11,30^. The *NR3C1* promoter region contains multiple methylation-sensitive cytosine-phosphate-guanine (CpG) dinucleotide repeats^11,31,32^, among these, the 1F region containing 47 CpG sites^12,33,34^.

*NR3C1* promoter methylation is responsible for different GR protein levels in various tissues^35,36^, such as the heart, kidney, lung, liver, skin, and especially the hippocampus^13,36–42^. Although it is not expressed in T-cells, it is expressed in B-cells and dendritic cells, homologous to the hippocampus; therefore, it can be evaluated in blood under conditions involving HPA axis changes^16,43^.

*NR3C1* promoter methylation may indicate lower mRNA levels and GR expression^13,44^. Thus, even high levels of peripheral cortisol are unable to bind to the cognate receptor to act as an HPA axis negative feedback mechanism, which could result in abnormal responses to stress^33,44^. On the contrary, since stress factors are associated with epigenetic changes, we hypothesized that social and behavioral factors may be associated with *NR3C1* gene methylation.

It is still unclear what different conditions might alter *NR3C1* promoter methylation, especially in broader and multifactorial systems. Thus, the present study aimed to analyze associations involving stressor factors such as socioeconomic status, health conditions, and lifestyle in relation to *NR3C1* gene methylation in adult individuals.

## Materials and methods

### Patient samples

This was a cross-sectional study carried out with users of the Brazilian Public Unified Health System (SUS) in a southeastern municipality (Alegre-ES), and was conducted between March 2017 and November 2018. The study population was composed of individuals living in urban and rural areas, and was approved by The Ethics Committee in Research with Humans of the *Universidade Federal do Espírito Santo* Health Sciences Center (CEP/CCS/UFES), under number 1,574,160, dated 6/6/2016. Individuals participating in the study signed written informed consent forms (ICFs). All methods were carried out in accordance with the Ethics Committee of Research with Humans.

### Population characteristics

This study was made up of a convenient sample of 386 individuals aged between 20 and 59 years who were users of the Brazilian Primary Health Care Units. Based on individual SUS registration forms, data were collected through individual interviews that evaluated socioeconomic, health, and lifestyle conditions. Low-income was defined as a per capita income/day less than US$5 (5 American dollars)^45^. Marital status, age, working conditions, and education (< 8 years, 8–11 years, and higher education) were also analyzed.

To assess habits and lifestyle features such as alcohol and tobacco consumption, in addition to leisure and physical activity, a structural questionnaire was applied based on the Research Directorate Work Coordination and Income Questionnaire for residents of households from the National Health Survey (NHS)^46^.

In this evaluation, the possible responses to alcohol and tobacco consumption were: (1) currently consuming, (2) used in the past, (3) never used, and for statistical analysis, responses were dichotomized into current consumption: no/yes. In addition, regarding leisure and physical activity, the possible answers for their activity were: (1) weekly, (2) biweekly, (3) monthly, and (4) not performed, dichotomized into yes/no activity.

In the same questionnaire, we addressed self-perceived health status in which the possible answers were: (1) very good, (2) good, (3) regular, (4) bad, (5) very bad, and were dichotomized into: good or very good/regular or poor health for statistical modeling.

Symptoms suggestive of depression were assessed using the Beck Depression Inventory-II (BDI-II)^47^. Values were categorized according to Gomes-Oliveira et al.^48^ considering normal or mild mood disorders (BDI-II < 17), and symptoms suggestive of depression (BDI-II ≥ 17).

Anthropometric assessment was carried out by qualified professionals using the Food and Nutrition Surveillance System (SISVAN)^49^, which collects information for directing public policies of the Brazilian Unified Health System (SUS). Information such as weight and height were collected, and from the obtained data, body mass index (BMI) was calculated and classified according to the World Health Organization^50^.

### Blood analysis

For analysis of cortisol and vitamin D levels and DNA methylation, 10 mL of peripheral blood was collected from patients by venipuncture after fasting for at least 8 h as instructed by community health agents (CHA). In a tube containing ethylenediaminetetraacetic acid (EDTA) anticoagulant, 3 mL of the sample was transferred for molecular analysis, and 2 mL into a tube containing NaF (sodium fluoride) anticoagulant for vitamin D analysis. The remaining blood was transferred to a tube without anticoagulant but containing a separating gel to obtain serum to determine cortisol levels. The tube contents were homogenized by inversion 5–8 times, and stored in refrigerated coolers over the range of − 2 to 8 °C. Blood samples for biochemical evaluation of cortisol and vitamin D levels were transported to Hermes Pardini Laboratory, Belo Horizonte, MG, while samples for DNA extraction were transported to the Biotechnology Laboratory, at the Center for Exact, Natural and Health Sciences, at the Federal University of Espírito Santo (CCENS/UFES).

Cortisol levels and vitamin D abundance were quantified by chemiluminescence, with reference values for morning cortisol levels of 6.7–22.6 μg/dL^17^. Results were classified as follows: low cortisol: < 6.7 μg/dL; Normal CORTISOL: 6.7–22.6 μg/dL; high cortisol: serum levels > 22.6 μg/dL. At the data analysis stage, the variable was dichotomized into high cortisol (≥ 6.7 μg/dL) and not high cortisol (< 6.7 μg/dL). Vitamin D levels were classified as deficiency: < 20 ng/mL, insufficiency: 20–29 ng/mL, and sufficiency: ≥ 30 ng/mL, and for statistical evaluation, these scores were dichotomized into insufficiency: < 30 ng/mL, and sufficiency: ≥ 30 ng/mL^20,51^.

DNA extraction was performed as described by Salazar et al.^52^. A NanoDrop 2000/2000c Spectrophotometer was used to verify DNA quality and concentration for further *NR3C1* methylation analysis.

### Quantitative pyrosequencing methylation assay

Of all samples with good DNA quality and concentration, a subsample of 285 patients was randomly assigned to undergo pyrosequencing methylation assays.

Sodium-bisulfite conversion of 1 μg of DNA was performed using the EpiTect Bisulfite Kit (*Qiagen*, Valencia, CA, USA), following the manufacturer’s recommendations. Pyrosequencing methylation assays were performed as previously described^53,54^.

PCR product quality was checked on 2% agarose gels using GelRed (*Uniscience*). Pyrosequencing was performed using a PSQ 96 ID Pyrosequencer (*Qiagen*, Valencia, CA, USA) with PyroMark Gold Q96 Reagent Kit (*Qiagen*), according to the manufacturer's protocol. All pyrosequencing conditions are listed in Table 1.

**Table 1:** PCR and Pyrosequencing primers and conditions.

PCR primer		Conditions		
Forward	5′-TTTTTTTTTTGAAGTTTTTTTA-3’	95 °C	(14′30″)	
Reverse	5′-BIOTIN-CCCCCAACTCCCCAAAAA-3’	94 °C	(30″)	
		50 °C	(30″)	45 cycles
		72 °C	(30″)	(410 bp)
		72 °C	(10′)	
		4 °C	Indefinitely	
**Sequencing primers**				
40 to 42 CpG	5′-AGAAAAGAAATTGGAGAAATT-3′			
43 to 47 CpG	5′-GTTTTAGAGAGATTAGGT-3′			
**Analyzed sequences**				
Seq 1	YGGTGGTTTTTTTAAYGTYGTTTTAATCGTGTTGATCAGTCGCTTA			
Seq 2	YGGTTTTYGTYGTTGTYGTYGTTAGTCAGTTCAGTCGTAGTCAGTCGTA			

A single pyrosequencing reaction was performed for each individual, and the reaction was evaluated for quality. The percentage of methylation was recorded for each CpG from 40 to 47, individually and on average, evaluated in PyroMark Q96 ID Software 2.5 version 2.5.10.7, using default software settings. In this study, we considered all methylation levels detected in pyrosequencing to classify individuals as methylated or unmethylated, when they presented methylation at any percentage above zero.

**Figure 1:**
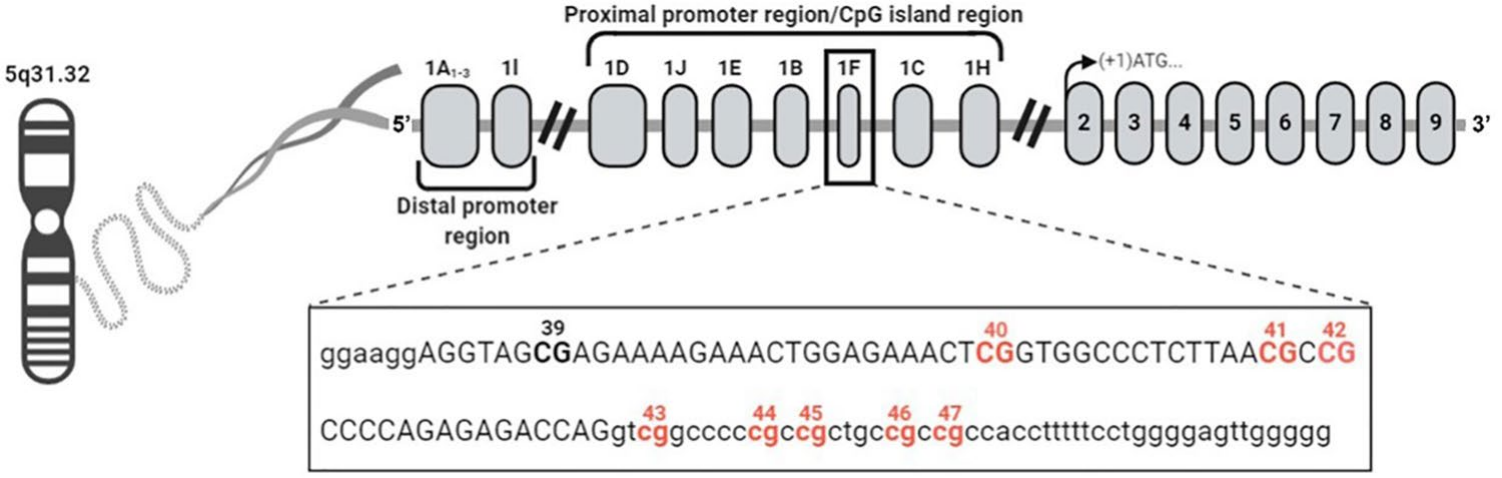
Promoter region of NR3C1 examined within this study. The CpGs studied (40-47) are represented in red and are also numbered. Lowercase nucleotides represent intronic regions, while uppercase nucleotides represent exon 1F. GenBank (NCBI—Access number: AY436590.1).

A representative scheme of the amplified 1F region of *NR3C1* and the eight CpGs site-specific analyzed using bisulfite-pyrosequencing assays are shown in Fig. 1.

### Statistical analysis

Data were analyzed using Chi-squared tests in a 2 × 2 contingency table and a statistical significance level of 5%. Quantitative data are expressed as median and interquartile ranges.

Results of quantitative analysis of *NR3C1* gene methylation did not follow a normal distribution, even after exponential conversion. In this manner, the methylation data were dichotomized. The mean methylation values of CGs segment from 40 to 47 were calculated for qualitative analysis such that values > 0 were categorized as methylated, and values equal to 0 were categorized as unmethylated. Then, applied bivariate analyses were performed using Poisson regression models with robust variance, with the dependent variable being methylation of the segment, and as explanatory variables socioeconomic aspects, health, and lifestyle, as well as suggestive symptoms of depression. After data characterization, independent variables were recategorized dichotomously according to the classifications in normal state/altered states before being included in the multivariate study model.

Predictive variables that achieved p values lower than 0.20 (p < 0.20) were inserted into the multivariate Poisson regression model with robust variance. The backward method was used, and those variables with less significance (greater p value) were removed one by one from the model. The procedure was repeated until all variables present in the model were statistically significant (p < 0.05). The Hosmer–Lemeshow test was used to verify the fit of the final model. The prevalence ratio (PR) with 95% confidence interval (95% CI) was used as an effect measure. For all analyses, a significance level of 5% was adopted. Statistical analyses were performed using SPSS software (v.13.0 for Windows) and Stata v.11.0.

## Results

### Socioeconomic profile

Our results were obtained from a total of 285 individuals evaluated by pyrosequencing of the *NR3C1* 1F region; 198 individuals did not show any percentage of methylation detected, whereas 87 exhibited measurable percentages of methylation. The median methylation level was 0.0 (0.0–12.9%). Table 2 presents a population description, which was divided into unmethylated and methylated. Individuals were predominantly female (80.6%), 75% lived with a partner, and the median age was 42.5 (33.7–52.0) years. Most of them had < 8 years of formal education (46.4%), low income (70.2%), did not drink, smoke, or perform physical or leisure activities.

**Table 2:** Sample characteristics according to methylation of 1F region *NR3C1* gene.

Characteristic	Total		Methylation				***p***
			No		Yes		
	N	(%)	N	(%)	N	(%)	
**Gender**							
Male	60	21.0	50	17.5	10	3.5	0.009*
Female	225	79.0	148	52.0	77	27.0	
**Age (years)**							
20–40	125	43.8	87	30.5	38	13.3	0.967
41–60	160	56.2	111	39.0	49	17.2	
**Marital status**							
Single	70	24.6	55	19.3	15	5.3	0.057
No single	215	75.4	143	50.2	72	25.2	
**Education**							
Basic education	240	84.2	167	58.6	73	25.6	0.926
Higher education	45	15.8	31	10.9	14	4.9	
**Working**							
Yes	151	53.0	103	36.2	48	16.8	0.623
No	134	47.0	95	33.3	39	13.7	
**Income**							
Non-low income (≥ $5.00/day)	81	28.4	58	20.4	23	8.0	0.623
Low income (< $5.00/day)	204	71.6	140	49.1	64	22.5	
**Tobacco consumption**							
No	261	91.6	176	61.8	85	29.8	0.014*
Yes	24	8.4	22	7.7	2	0.7	
Alcohol consumption							
No	201	70.2	125	43.9	75	26.3	0.000*
Yes	84	29.8	73	25.6	12	4.2	
**Weekly drinking**							
< 7 drinks	64	76.2	53	63.1	11	13.1	0.438
> 7 drinks	20	23.8	18	21.4	2	2.4	
**Physical activity**							
Yes	95	33.3	69	24.2	26	9.1	0.413
No	190	66.7	129	45.3	61	21.4	
**Leisure activity**							
Yes	132	46.3	97	34.1	35	12.3	0.172
No	253	53.7	101	35.4	52	18.2	
**Self-rated health**							
Good or very good	145	50.9	105	36.8	40	14.0	0.273
Regular or poor	140	49.1	93	32.6	47	16.5	
**Depression**							
BDI-II < 17	194	68.1	136	51.3	58	21.9	0.142
BDI-II ≥ 17	71	24.9	43	16.2	28	10.6	
Not available^#^	20	7.0					
**Body mass index—BMI**							
No overweight	96	33.7	59	20.7	37	13.0	0.036*
Overweight	189	66.3	139	48.8	50	17.5	
**Vitamin D**							
Sufficiency	264	92.6	185	64.9	79	27.7	0.434
Deficiency	21	7.4	13	4.6	8	2.8	
**High cortisol levels**							
No	270	94.7	185	67.0	85	30.8	0.107
Yes	6	2.1	6	2.2	0	0.0	
Not available^#^	9	3.2					
Total	285	100.00	198	69.50	87	30.50	

*BDI-II* Beck Depression Inventory-II, *BFIS* Brazilian Food Insecurity Scale, *FNS* Food and Nutrition Security, *FNiS* Food and Nutrition Insecurity, *BMI* Body Mass Index. ^#^Not available (not considered in the statistical calculations). Categorical variables presented in relative (%) and absolute (n) frequencies. Quantitative variables presented in medians and interquartile ranges (IR), according to normality (Kolmogorov–Smirnov test); * p value: Mann–Whitney U or chi-square, at 5% significance (p < 0.05).

### Methylation analysis of *NR3C1*

The average methylation index was calculated from CpG sites methylation percentages. These percentages were dichotomized into unmethylated (0% methylation) and methylated (values > 0% methylated).

Bivariate Poisson regression showed associations between methylation profiles and gender (p = 0.047), alcohol consumption (p < 0.001), depression (p = 0.022), body index (p = 0.017), and cortisol levels (p < 0.001) (Table 3).

**Table 3:** Bivariate Poisson regression analysis with robust variance for *NR3C1* 1F region methylation.

Characteristics	Methylation		
	(CpG 40–47)		
	PR	95% CI	p
**Gender**			
Male	1.8	1.00–3.41	**0.047**
Female			
**Age (years)**			
20–40	1.0	0.70–1.47	0.938
41–60			
**Marital status**			
Single	1.6	0.95–2.63	0.073
Not single			
**Education**			
Basic education	0.9	0.55–1.54	0.779
Higher education			
**Working**			
Yes	0.9	0.67–1.43	0.785
No			
**Income**			
Non-low income (≥ $5.00/day)	1.2	0.77–1.80	0.443
Low income (< $5.00/day)			
**Tobacco consumption**			
No	0.2	0.69–1.04	0.058
Yes			
**Alcohol consumption**			
No	0.3	0.16–0.53	** < 0.001**
Yes			
**Physical activity**			
Yes	1.1	0.76–1.72	0.500
No			
**Leisure activity**			
Yes	1.2	0.87–1.87	0.196
No			
**Self-rated health**			
Good or very good	1.3	0.90–1.91	0.147
Regular or poor			
**Depression**			
BDI-II < 17	1.5	1.06–2.27	**0.022**
BDI-II ≥ 17			
**Body mass index—BMI**			
Not overweight	0.6	0.44–0.92	**0.017**
Overweight			
**Vitamin D**			
Sufficiency	1.2	0.69–2.40	0.413
Deficiency			
**High cortisol levels**			
No	2.2^–9^	1.11^–9^–4.65^–9^	** < 0.001**
Yes			

Bold values indicate significance result.

*PR* prevalence ratio, *95% CI* confidence interval, *p* p value.

From variables in the Bivariate Poisson regression with values p < 0.20, the multivariate model was designed. The results showed that methylation is associated with alcohol consumption, depression, BMI, and high cortisol levels (Table 4).

**Table 4:** Multivariate Poisson regression analysis with robust variance for methylation of *NR3C1* 1F region.

Characteristic	Methylation		
	(CpG 40–47)		
	PR	95% CI	p
**Alcohol consumption**			
No			
Yes	0.30	0.16–053	** < 0.001**
**Depression**			
BDI-II < 17			
BDI-II ≥ 17	1.55	1.07–2.24	**0.018**
**Body mass index—BMI**			
Not overweight			
Overweight	0.66	0.46–0.95	**0.017**
**High cortisol levels**			
No			
Yes	0.09^–5^	3.8^–7^–1.9^–6^	** < 0.001**

Bold values indicate significance result.

*PR* prevalence ratio, *95% CI* confidence interval, *p* p value.

As observed in Table 4, alcohol consumption was associated to 70% decrease in the prevalence of methylation (1–0.30 × 100); being overweight showed a 34% lower prevalence of methylation than not being overweight (1–0.66 × 100), and high cortisol was associated with a lower prevalence of methylation, however with a measure of low effect PR = 0.09^–5^. In contrast, depression exhibited the opposite effect, being directly related to methylation with prevalence of 55% higher than non-depressed individuals (1.55 times more prevalent).

The final model was statistically significant (p < 0.001), presenting a pseudo r^2^ = 0.0759 and, after adjustment by Hosmer and Lemeshow, showed good adherence (p = 0.99).

## Discussion

This study presents individuals with low income, low education, and predominantly of female gender. Our goal was to establish a broader assessment of factors related to *NR3C1* gene methylation, such as socioeconomic aspects, health, and lifestyle.

We have shown that alcohol consumption, overweight, and high cortisol levels are related to *NR3C1* non-methylation, while depression is related to its methylation. Argentieri et al.^16^ presented a series of studies that related hyper- or hypomethylation with specific CpG methylation of this gene.

Few studies have evaluated the relationship between methylation patterns and alcohol consumption. It was expected that alcohol as a stressor factor could stimulate the HPA axis by increasing cortisol levels and *NR3C1* methylation. Gatta et al.^24^ reported hypermethylation of *NR3C1* exon 1H in the prefrontal cortex of individuals with alcohol abuse disorders compared with that in a control group. However, our data showed low levels of *NR3C1* 1F region methylation. Corroborating our findings, Dogan et al.^25^ showed a relationship between alcohol consumption and decreased levels of *NR3C1* methylation in a 64-patient cohort.

Studies have demonstrated that alcohol consumption may lead to HPA axis alterations, with glucocorticoid release modifications^55,56^. Here, we showed association between alcohol consumption and *NR3C1* methylation, which indicated that alcohol-mediated modulation of the HPA axis may occur through epigenetic changes.

Although self-reporting of alcohol consumption was insufficient to control its effects on *NR3C1* methylation, our finding is relevant because alcohol represents a large fraction of the drugs consumed by the world population, and the epigenetic effects induced by its consumption are still little known.

Our analysis showed an association between suggestive symptoms of depression, as evaluated by Beck scores ≥ 17, with increased methylation at *NR3C1* 40–47 1F CpGs sites. Other authors have associated methylation alterations in the 1F region CpG 36–39 sites with depressive status in adolescents^57^ and in CpGs 36–44 on maternal exposure to gestational stress and depression in children^58^.

In addition, other authors have studied the 1F region, showing hypomethylation involving CpGs 35–47, with hypomethylation specifically of CpG 43 associated with depression^59^. There are also studies addressing the 1F region showing hypomethylation of CpGs 35–39 in individuals with depression^19^. However, the present study provides new information that individuals with depressive symptoms, and frequent use of the public health system, exhibit methylation of the *NR3C1* 1F region.

In this manner, it is possible that *NR3C1* methylation is associated with depression, in that *NR3C1* gene methylation may indicate lower mRNA levels and GR expression, leading to imbalanced HPA axis modulation, which could result in abnormal responses to stress, and increase susceptibility to depression^33^^,^^44^^.^

Furthermore, high cortisol levels were associated with non-methylation, but with a very low prevalence ratio (Table 4). High levels of cortisol have already been related to *NR3C1* gene methylation in maternal and postnatal gestational exposure to childhood stress^14,33,60^.In our study, only 8 individuals presented high cortisol levels, while the others had normal or low levels, but which may not be representative.

On the contrary, multivariate analysis of risk factors showed that being overweight is associated with non-methylation, with a prevalence ratio of 0.67, indicating that being overweight reduces the prevalence of methylation by 33%. Chronic stress has previously been related to increased cortisol levels, leading to weight gain^61^. Excess weight is also related to chronic inflammation through NFκB pathways; however, no association between overweight and methylation status has been previously reported for the *NR3C1* gene^62^. It is possible that hypomethylation of this region may be related to low-grade inflammation, a feature of the overweight state^63^.

Although it was a cross-sectional study, and it is not possible to establish any causal relationships, this study is relevant as it revealed a direct or inverse association between methylation, alcohol consumption, overweight, and high cortisol levels related to *NR3C1* non-methylation, whereas depression was related to methylation.

Thus, we suggest that habits, lifestyle, and health status may influence *NR3C1* gene regulation via methylation. Relationships involving genotype, environment, and phenotypic outcomes may be more refined than previously thought, depending on specific stressful events that can result in unique clinical consequences.
